# Mobilising Zionist and philo-Semitic sentiments through melodrama: Brazilian biblical telenovelas in the production of a neo-Pentecostal political culture

**DOI:** 10.1080/09637494.2025.2526999

**Published:** 2025-10-14

**Authors:** Manoela Carpenedo

**Affiliations:** Faculty of Religion Culture and Society, University of Groningen, Groningen, the Netherlands

**Keywords:** Christian Zionism, philo-Semitism, religion and media, religion and telenovelas, media and politics, dispensationalism

## Abstract

Biblical telenovelas have been extensively produced by one of the world’s largest neo-Pentecostal churches, the Brazilian Universal Church of the Kingdom of God (UCKG). Since 2010, the UCKG’s TV network has been broadcasting and exporting these epic biblical series, which have played a significant role in solidifying a unique neo-Pentecostal melodramatic culture. This contribution delves into the telenovela *Apocalypse* (of 2017–2018 and broadcast again in 2024 during the war in the Middle East) to demonstrate how biblical telenovelas can foster political sentiments. Research in the field of media and religion has underscored how media can shape religious imagination, allow individuals to experience transcendence, and create alternative avenues for religious practice due to its meaning-making capabilities. Furthermore, studies on Pentecostal telenovelas have shed light on the evangelising potential of the genre and its significance in the public domain. However, there is a gap in understanding how these melodramatic narratives not only activate religious imagination but also political inclinations. This contribution seeks to fill this gap, examining how everyday politics, philo-Semitic, and Zionist inclinations are nurtured through the UCKG’s Biblical telenovelas.

## Introduction

Biblical telenovelas have been extensively produced by one of the world’s largest neo-Pentecostal churches, the Brazilian Universal Church of the Kingdom of God (UCKG, *Igreja Universal do Reino de Deus*). The church operates in 135 countries, boasting a membership of around seven million people in Brazil and 2.9 million in other nations. The UCKG owns a TV network, Record, which ranks as the second most watched and profitable TV channel in Brazil. TV Record’s line-up is mixed and features secular TV programmes, as well as numerous televangelism shows and a diverse range of religious content. Since 2010 Record has ramped up the production, broadcasting, and exportation of epic biblical series on a global scale. For example, in 2015 Record aired the telenovela *Ten Commandments*. This series was so compelling that one of its episodes became the most-watched programme on open TV in Brazil (Stycer 2015). Following this triumph, the TV network has continued to invest heavily in the biblical telenovela format, cementing a distinct neo-Pentecostal melodramatic culture in the country and beyond.

The rise of Pentecostal evangelicalism in Brazil offers valuable insights into the growing interest in biblical telenovelas. This religious group has increasingly challenged the longstanding dominance of the Catholic Church, expanding by 61% over the past decade (IBGE 2010). While Pentecostal evangelicals constitute a significant audience for these cultural productions, biblical narratives also appeal to a broader spectrum of Brazilians, including Catholics and individuals without any religious affiliation (Gomes 2017). The remarkable success of Brazilian biblical telenovelas raises compelling questions regarding the relevance of the biblical genre in popular culture and the role of media in shaping religion within the public domain.

Research in the field of media and religion has highlighted how media can shape religious imagination (Hjarvard 2011), allow individuals to experience transcendence through ‘sensational forms’ (Meyer 2009), and create alternative avenues for religious practice owing to media’s meaning-making capabilities (Hoover and Echchaibi 2021). Additionally, studies on Pentecostal telenovelas have illuminated the evangelising potential of the genre and its prominence in the public domain (Pype 2012). However, a gap remains in understanding how these melodramatic narratives not only stimulate religious imagination and emotions but also embed political inclinations. This contribution seeks to bridge this gap, exploring how certain political perspectives are cultivated through the UCKG’s biblical telenovelas. It examines the telenovela *Apocalypse* (2018), produced by Record TV, to illustrate how this TV show, grounded in Brazilian neo-Pentecostal culture and certain interpretations of dispensational theology, can engender not only a particular religious moral programme anchored in testimonies, prayer, and Bible study but also Zionist and philo-Semitic sentiments.^1^ In doing so, it both integrates the political dimension to the analysis of religious telenovelas and underscores the significance of Zionism and philo-Semitism in shaping contemporary neo-Pentecostal political culture.

In the first section of the contribution, I delve into current debates surrounding religion, media, and political culture. Subsequently, I provide context for Pentecostal telenovelas. After addressing methodological considerations and introducing the case study, I examine how biblical telenovelas have shaped a distinct neo-Pentecostal political culture and ethos. Finally, I explore the transmission of Zionist and philo-Semitic sentiments through these melodramatic narratives. I argue that Brazilian biblical telenovelas contribute to the construction of a neo-Pentecostal political culture that promotes not only a religious moral programme but also Zionist and philo-Semitic sentiments.

## Religion, media, and political mobilisation

Scholars dedicated to the sociology and anthropology of religion have highlighted the interconnectedness of religion and media (Meyer and Moors 2005). Throughout history, communication about the sacred was made possible through different media, such as written texts, images, icons, architecture, and music (Stolow 2005). Today, religion is not only produced but also made visible in the public sphere through media forms. Through media, individuals can experience transcendence through its ‘sensational forms’ (Meyer 2009). At the same time, Plate (2003) reminds us that media of communication are not merely empty receptacles connecting the addresser and addressee. Instead, they are dynamic entities that actively shape and reshape the world. As Plate states, ‘religions and cultures do not merely *use* media, but instead are *used* by media and [are] created by them’ (2003, 10)

Religious groups are aware of the symbiotic relationship between media and religion and have leveraged various media forms to disseminate their beliefs and establish cultural networks. Televangelism in the United States serves as a prime example. Televangelists’ goals extend beyond preaching the gospel globally, as they also aim to build a religious community. Alexander (1997) observes that American Christian viewers engage actively with these broadcasts, seeing themselves as participants in a spiritual battle against the secular values prevalent in mainstream society. They view their role as divine agents on earth, charged with the mission of transforming a world they believe to be under satanic influence. Televangelism thus educates its audience on social and political matters and encourages them to take an active role in politics, reflecting their commitment to their faith and its societal applications (Alexander 1997). A notable example of televangelism being employed for political means is seen in the case of televangelist Jerry Falwell, who founded the Moral Majority organisation. This pioneering religious right organisation mobilised Christian voters in the eighties, playing a significant role in Ronald Reagan’s 1980 election (Williams 2010). The Moral Majority was marked by its social and economic conservatism, and it strongly emphasised moral issues, taking stands against abortion, LGBTQ+ rights, and the Equal Rights Amendment (Williams 2010).

In Brazil, televangelism evolved from a strong tradition of press and radio religious outreach among evangelicals (Cunha 2012). While the virtual sphere and the internet are becoming increasingly accessible to many Brazilians, radio and TV remain the most widespread media, reaching broader geographic areas in the country (Lima 2005). Today, traditional Pentecostal churches, such as the Assemblies of God (*Assembleia de Deus*) and the prominent UCKG, as well as the Catholic Church – through its charismatic renewal – heavily rely on their respective TV channels for proselytisation.

Reflecting this urge, the UCKG purchased the TV Record network in 1989. Mixing secular and religious TV shows in its line-up, TV Record has been the second-most watched TV channel in Brazil since 2007 (Pantoja and Lisboa 2020). The UCKG uses its television network to broadcast a variety of programming, including news, sports, and reality shows, while maintaining a focus on family values. Central to its mission is televangelism, as evidenced by shows like *You Can Speak and I Will Listen*, which combines journalistic reporting with neo-Pentecostal interpretations, addressing social issues such as violence and drug abuse (Pantoja and Lisboa 2020). Additionally, segments featuring personal testimonies are key, where individuals recount their transformative experiences after converting to Christianity, showcasing the stark contrast between their troubled pasts and their current, happier lives as born-again Christians (Francisco 2011).

As observed in the North American context, Christian networks and their leaders can be deeply involved in political issues and national elections. Edir Macedo, the leader of UCKG and owner of TV Record, has actively leveraged his religious influence to support his political views and preferred politicians. Although Macedo initially opposed left-wing presidential candidates following Brazil’s re-democratisation in 1989 (Mariano 2014), he shifted his position in 2002, aligning himself with the left politician Luiz Inácio Lula da Silva and the Workers’ Party (Cruz 2009). However, his position shifted again and he supported the extreme right-wing candidate Jair Bolsonaro in the 2018 and 2022 presidential elections (Mariano and Gerardi 2020). Macedo contended that the agenda championed by Lula and the left clashed with evangelical beliefs and biblical principles (Mariano and Gerardi 2020). Notably, in the 2018 elections there was evident collaboration between UCKG and Bolsonaro, with the latter being frequently invited for interviews and appearances on TV Record shows (F. R. C. Oliveira and Martins 2021). This support stemmed from UCKG’s stance on traditional family values. Bolsonaro positioned himself in the Brazilian electoral landscape as a champion of morality and family values, which manifested as resistance to the progression of women’s and LGBTQ+ rights. During Bolsonaro’s administration, TV Record was the network that received the most federal financial incentives (Prazeres 2019). In the 2022 elections, Macedo supported Bolsonaro again (Benício 2022).

These examples underscore the significance of examining how political sensibilities are mobilised by televangelism and its religious leaders. While it is essential to understand how political messages and electoral endorsements are openly conveyed by Christian networks, this contribution goes a step further in the debate by exploring how political imaginaries can be manufactured through fictional productions such as telenovelas.

## Brazilian telenovelas and Pentecostal melodrama

Telenovelas are instrumental in shaping cultural narratives in Brazil, as well as throughout Latin America and other regions. While they share some resemblance to North American soap operas, Brazilian telenovelas diverge in both structure and storytelling approach. Unlike the open-ended nature of North American soaps, which can run indefinitely and are often broadcast during daytime to cater to a predominantly female audience, Brazilian telenovelas are designed as finite series. Airing daily in prime-time slots, they span around 200 episodes and form complete narratives with defined beginnings, middles, and endings, wrapping up within several months. These telenovelas capture the highest viewership ratings on Brazilian television, with storylines that present clear moral dichotomies and dramatic character arcs, such as a daughter discovering the truth about her mother’s identity or a destitute woman overcoming adversity to eventually marry a wealthy man.

Studying the cultural impact of Brazilian telenovelas over decades, Maria Immacolata Vassallo de Lopes (2009) is convinced that telenovelas have become a significant form of narrative about the nation, offering a way for viewers to engage with and participate in an imagined national identity. According to Lopes, audiences incorporate elements from the shows into their daily lives, such as using slang and mannerisms from characters, naming children after popular characters, and even naming businesses after telenovelas. According to Lopes (2009), when a telenovela captures the nation’s attention, it has the potential to influence and express the nation’s collective imagination and its sense of identity. Despite being melodramatic and open to interpretation, these representations are widely accepted as plausible, legitimate, and credible, making them a central part of cultural life (Lopes 2009).

This contribution draws on Lopes’ considerations and examines telenovelas as a communicative resource in shaping a ‘narrative of the nation’. Telenovelas hold significant cultural value in Brazil, functioning not only as a form of entertainment but also as a medium through which Brazilians engage with gender roles and navigate consumerism (Hamburger 2005, 2011). Additionally, they play a crucial role in the construction of collective imaginaries and national identities (Martín-Barbero 2004). Brazilian telenovelas have enjoyed widespread success beyond Brazil’s borders since the 1970s, initially reaching Portugal and other Latin American countries. Over time, they have expanded into diverse regions, including Cuba, China, former USSR countries, and western nations such as the United States, Italy, and France (Hamburger 2011).

Religion holds a significant place in Latin American telenovelas. Given the stark delineation between good and evil in telenovela plots, combined with a clear moral narrative that commends virtues and reprimands vices, telenovelas can act as moral guides for audiences transitioning from predominantly Catholic societies to more secular ones (Rey 2020). Drawing inspiration from Catholic themes such as guilt, suffering, and purification, telenovelas can also convey theological messages (White 2007). More recently, with the Pentecostal boom in Latin America and beyond, Catholic imaginaries are gradually being replaced by Pentecostal evangelical ones.

For instance, Katrien Pype’s 2012 study delves into Congolese Pentecostal television dramas, coined as ‘Pentecostal melodramas’. These shows intertwine Pentecostal beliefs and church teachings within their narratives, portraying the everyday challenges faced by ordinary people. The plots often revolve around characters combating demonic influences that cause misfortune, poverty, infertility, and illness in their lives. Through Pentecostal prayer and guidance from pastors, these forces are overcome, illustrating a path to ‘the good life’. The melodramas not only spread the word of Pentecostalism but have also contributed to forming a common Christian identity in Kinshasa.

The research object of this study is one of the neo-Pentecostal biblical telenovelas of UCKG’s TV Record, entitled *Apocalypse*. These productions blend the Latin American telenovela format, typically comprising approximately one hour of content and spanning about 200 episodes aired daily, with the evangelising and pedagogical objectives outlined by Pype (2012). It is important to stress that the telenovelas produced by TV Record do not have the same traction and viewership ratings as the rival TV Globo, which is the leading open TV channel in Brazil. TV Record began producing brief biblical teleseries in the 1990s, a trend that gained momentum starting in the 2010s with releases such as *Esther* (2010), *Samson and Delilah* (2011), and *Joseph of Egypt* (2013). *Ten Commandments* (2015) marked the inaugural fully-fledged biblical telenovela, setting the stage for subsequent series like *Promised Land* (2016), *The Rich Man and Lazarus* (2017), the focus of this study, *Apocalypse* (2017–2018), and *Kings* (2023–2025).

These biblical melodramas are defined by their emphasis on the four Gospels and the book of Psalms, as well as narratives from the Old Testament. These television productions have brought about a significant transformation in the entertainment landscape of Pentecostal groups in Brazil. Most Pentecostal and neo-Pentecostal churches encourage their members to abstain from secular media, especially mainstream telenovelas, and non-gospel music, which are often deemed as immoral and vulgar. Consequently, the advent of these biblical telenovelas fills a void in entertainment for these groups, allowing them to enjoy religious narratives without compromising their beliefs and values.

Although these biblical productions are often criticised by some conservative religious groups for their colloquial and popular tone – viewed as a vulgarisation of important biblical and religious themes through excessive dramatisation and romanticisation – their dramatic appeal allows them to reach a wider audience, including Catholics and non-religious individuals not necessarily affiliated with Pentecostalism (Gomes 2017). Undertaking qualitative research on the reception of biblical telenovelas among Pentecostal believers and the non-religious, Santos and Malta (2024) found that for Pentecostal viewers, these biblical telenovelas offer family-friendly content and convey Christian values in an entertaining manner. Non-religious audiences, who may have never had the opportunity to engage with the Bible, find watching these telenovelas to be a way of learning about biblical narratives and morality (Santos and Malta 2024). Moreover, for non-believers and Catholic believers, audiences are drawn to fictional biblical content primarily because it offers a sense of wellbeing and spiritual uplift, rooted in its connection to the Bible and its premise of ‘elevating the spirit’. In contrast, non-religious telenovelas – which often explore themes such as violence, sex, and prostitution – are perceived as heavy and emotionally taxing, making it difficult for viewers to relax or feel at peace (Santos and Malta 2024). Therefore, the consumption of these audiovisual products by various religious and non-religious groups highlights the eclectic nature of religious media in contemporary contexts, as different groups have the ability to reinterpret and derive diverse meanings from these productions.

## Neo-Pentecostal culture, philo-Semitism, and Christian Zionism

The growing popularity of biblical telenovelas is closely linked to the rise of neo-Pentecostal culture in the Global South. One noteworthy aspect of this expanding culture is its political dimension. In contrast to first-wave Pentecostals in Latin America, who believed that politics was too worldly and tainted to directly engage with, the subsequent wave of Pentecostals – neo-Pentecostals – assert that Christianity should exert a growing political influence in the world (Guadalupe 2022). Neo-Pentecostal believers are encouraged to gradually work towards advancing the gospel and its values worldwide to transform the world into a better place. This mandate encourages a very active political role for these groups and clarifies the increasing political activism undertaken by Pentecostals in Brazil.

Another aspect of this political shift is the growing adoption of philo-Semitism and the increasing support for the Zionist cause among Brazilian Pentecostals (see Carpenedo 2018, 2021; Freston 2020; Machado, Mariz, and Carranza 2021). See also Freston et al. in this collection). Philo-Semitism and Christian Zionism within Brazilian Pentecostal evangelicalism draw motivation from certain biblical writings, such as Genesis 12:3,^2^ which convey the idea that God will bless nations that protect Israel and curse those that oppose it. Contributing to this shift, many Pentecostal groups have been incorporating elements of dispensational theology into their teachings over the past decade. For instance, the Assemblies of God – one of the largest Pentecostal denominations with over 22.5 million members in Brazil – adopted dispensationalist teachings through the missionary work of Lawrence Olson. His book *O Plano Divino Através dos Séculos* (‘The Divine Plan Through the Ages’) (Olson 1981) played a key role in introducing these ideas (D. M. Oliveira et al. 2016). Dispensationalism is explicitly affirmed in the Assemblies of God’s declaration of faith (Santin 2022).

The dispensationalist biblical narrative consists of a series of distinct eras reflecting God’s interaction with humanity, known as ‘dispensations’. According to this belief, the seventh and final dispensation, the ‘Age of the Kingdom’, will signal the end times. A period known as the Great Tribulation is predicted to occur just before the Age of the Kingdom’s onset, featuring a seven-year rule by the antichrist. During this time, Charismatic evangelical Christians believe they will be taken to heaven during the Rapture, while non-Christians, including Jews, will face the antichrist’s influence on earth. It is also expected that a portion of the Jewish population will embrace Christianity, and Jesus will return to defeat the antichrist. Following this, Jesus is prophesied to govern the world from Jerusalem throughout the millennium before the final judgement and to establish a new earthly paradise.

This theological viewpoint diverges significantly from mainstream supersessionist Christian teachings, which hold that Christians have replaced the Israelites as God’s chosen people. Supersessionist ideas have been widely debated by Christian theologians, particularly in light of their historical relationship to the Holocaust. For example, Rosemary Radford Ruether (1996) argues that supersessionist theology portrayed Judaism as spiritually inferior and outdated. This framework not only fostered anti-Jewish sentiment but also actively legitimised social and political discrimination, contributing to the ideological conditions that made the Holocaust possible. The Pentecostal theology embraced in Brazil, however, diverges from both classical supersessionism and its critiques by post-Holocaust theologians. Rather than viewing the Church as the new Israel, many Pentecostal groups adopt versions of a dispensationalist framework. Dispensationalism maintains a clear distinction between Israel and the Church, seeing them as two separate entities in God’s redemptive plan. God’s promises to Israel – such as land, nationhood, and blessing – are viewed as eternal and irrevocable, and thus still applicable to ethnic Israel, not transferred to the Church. In this view, the Church is a spiritual body, uniquely created for the current dispensation to proclaim the gospel and prepare believers for a heavenly destiny. It does not inherit Israel’s national promises and is not considered the fulfilment of Old Testament prophecies – a role that remains reserved for the ethnic Israel.

Those who adhere to the dispensational viewpoint within Christianity believe that the return of Jews to the Holy Land and their subsequent conversion to Christianity are crucial prophetic events necessary for the establishment of the Kingdom of God. This culminates in Jesus’ reign in heaven on earth. According to this perspective, without the return of Jews to Israel, there will be no second coming of Christ and, consequently, no resurrection or promise of eternal life in heaven on earth for Christians. This perspective helps us to understand the strong political support and financial assistance to Israel being organised by some Christian networks embracing dispensational teachings.

While it remains important to acknowledge the influence of dispensationalism in shaping Christian support for Israel – particularly within Brazilian Pentecostalism – I agree with Westbrook’s (2014) critique within the US context, that we must look beyond theology alone. Support for Israel increasingly transcends doctrinal frameworks and becomes entangled with nationalistic sentiments, reflecting a broader shift in how nationalism is expressed in a globalised world, shaping both religious practices and political alignments (Spector 2009; Westbrook 2014).

In Brazil, this unconditional Christian support for Israel manifests in various ways, especially through social media. For instance, after the 7 October 2023 armed incursion by Hamas and several other militant groups from Gaza into Southern Israel, numerous Brazilian Pentecostal pastors and evangelical leaders took to social media and organised public demonstrations to express strong support for Israel. This surge of solidarity was evident across various platforms and events. Evangelical social media channels were inundated with images of the Israeli flag and messages of unwavering support. This online activity mirrored the community’s theological beliefs, viewing the conflict through a prophetic lens. Another example were mass rallies organised in June 2024, during which over 40,000 Brazilian Christians convened in Manaus for a rally organised by the Brazilian branch of the International Christian Embassy Jerusalem (ICEJ). The event featured prayers for Israel and speeches highlighting the importance of standing with the Jewish state during times of conflict (ICEJ 2024). Moreover, during the March for Jesus in São Paulo in May 2024, participants prominently displayed a massive Israeli flag, symbolising the evangelical community’s support for Israel. This public display underscored the political and religious alignment between Brazilian evangelicals and the State of Israel (France 2024).

This contribution expands the discussion by exploring how dispensationalist inclinations can be translated into popular cultural forms, such as biblical telenovelas. In the US context, Daniel Hummel (2023) coined the term ‘pop dispensationalism’ to describe a simplified, media-driven version of dispensationalist theology that gained widespread popularity among North American evangelicals from the mid-twentieth century onward. This form of theology distilled complex eschatological ideas into emotionally gripping narratives, often centred on themes of apocalyptic urgency – such as the imminent Rapture, the rise of the antichrist, and prophetic interpretations of global events, especially in the Middle East. Fuelled by consumer culture and mass media, pop dispensationalism thrived through best-selling books and films, with the *Left Behind* novel series (1995–2007) and subsequent film adaptations of 2000, 2002, and 2005 serving as a prime example. According to Hummel, this model proved highly adaptable across denominations and sociopolitical contexts. Although it had a significant impact on shaping evangelical support for Israel and US foreign policy, pop dispensationalism began to decline in the 2000s due to repeated failed prophecies and growing theological criticism (Hummel 2023).

This study recognises the relevance of pop dispensationalism as a cultural and political force and analyses how its ideas have been adapted, localised, and popularised within Brazilian pop culture – particularly through biblical telenovelas that mobilise theology to engage with both political discourse and global religious imaginaries.

## Methodological considerations

Examining the way media production can influence political tendencies has become crucial. Television plays a pivotal role in social dynamics, circulating meanings and offering popular entertainment through its programmes (Fiske 2011). TV shows, like telenovelas, are integral to social and discursive practices not only in Latin America but also globally, reflecting the beliefs and values of their respective societies. To explore how philo-Semitism and Christian Zionist views are portrayed in biblical telenovelas, I focused primarily on *Apocalypse* – the first biblical telenovela by Record TV set in modern times, marked by clear dispensationalist and Christian Zionist themes, and aired between 2017 and 2018, finishing shortly before the 2018 elections that brought Bolsonaro to power. I watched all 155 one-hour episodes and conducted a detailed textual analysis of its religious and political depictions. Textual analysis allows us to interpret films, TV shows, and magazines as ‘cultural texts’, providing insights into how societies and individuals perceive their surroundings (McKee 2003).

This textual analysis is guided by the principles of cultural studies that emphasise how wider social processes of power, ideology, and oppression influence cultural production (Lynch 2009). From this viewpoint, understanding the creation of television shows and the meanings they convey requires a deep comprehension of discursive dynamics. Anchored in the Foucauldian tradition, this research posits that discourse is a socially constructed language or representational system that consistently communicates meanings about certain subjects. In other words, discourse is also historically contingent, produces knowledge and meaning, and reflects power dynamics (Foucault 2013). Discourses are neither individual creations nor top-down impositions; they are socially constructed at grassroots levels. They play a role not just in the creation of texts and media but also in shaping our understanding of social experiences.

This study considers these debates and examines how the biblical telenovela emerges as a culturally salient category able to produce and reproduce certain discourses. My analysis highlights the formation of a neo-Pentecostal political culture that includes the dissemination of a religious moral programme based on testimonies, prayer, and Bible study, as well the popularisation and normalisation of philo-Semitic and Christian Zionist discourses.

## Case study

The focus of this study is the telenovela *Apocalypse*, which originally aired between 2017 and 2018, was broadcast again from April to September 2020 (during the COVID pandemic), and most recently aired again from May to October 2024 (amid the war in the Middle East). Despite being a significant financial investment for TV Record, with production costs reaching $150,000 per episode (over 23 million dollars in total), the telenovela did not achieve the anticipated success. In its early weeks, it garnered 13 audience points, ultimately averaging 8 points overall (VEJA 2018). While it did not achieve the same level of success as other biblical telenovelas – such as *The Ten Commandments*, which averaged 20 rating points and peaked at 31, becoming one of the most-watched TV shows in Brazil in 2015 – *Apocalypse* still attracted attention. The show’s contemporary setting, which diverged from the ancient type of biblical format, allowed it to present a clear Pentecostal moral programme. This enabled the telenovela to embody a modern neo-Pentecostal culture and offer screen-based interpretations of eschatological themes very dear to neo-Pentecostals. Moreover, the fact that this telenovela was rebroadcast by the UCKG-owned network both during the COVID-19 pandemic and amid the current escalation of conflicts in the Middle East is significant, as it suggests an intentional effort by the network to frame these events through apocalyptic tropes – linking war and disease to the end times in ways that reinforce the doctrinal themes of the plot.

The telenovela draws inspiration from the biblical book of Revelation and interprets this book with a futuristic lens, positing that it unveils events of an imminent future. Inspired by dispensational teachings, the telenovela is focused on the period of the ‘Great Tribulation’, where the antichrist reigns for seven years, and good Christians are taken up to heaven during the Rapture while others endure tragic events.

Structured in three distinct temporal arcs, the telenovela melodramatically depicts the events of the end times. The narrative begins with the conception and early life of the apocalyptic figure, the antichrist. Ricardo Montana, the antichrist, is the product of a tumultuous relationship between a former Orthodox Jewish woman, Débora Koheg, and an Italian millionaire, Adriano Montana. Raised amidst familial strife in Rome, Ricardo is mentored by the ‘Sacred Father’ of the ‘Church of the Sacred Light’, symbolising the Roman Catholic Church. As an adult, Ricardo Montana emerges as a significant business and political figure. He methodically fulfils the actions attributed to the antichrist based on dispensationalist interpretations. As the global supreme leader, Ricardo constructs the third temple in Jerusalem and initiates a deceptive three-year global peace that soon devolves into widespread wars due to his insatiable lust for power. The narrative also sees Ricardo’s death and resurrection over three days, his enforcement of the beast’s mark (a microchip) in the global population, and his brutal persecution of those who refuse to worship him. Opposing the antichrist’s tyranny are the protagonists, Benjamin Gudman and Zoe Santero, devout neo-Pentecostals who spearhead the resistance against Ricardo.

The telenovela was filmed in various locations and featured a multitude of characters and different dramatic storylines. One storyline is set in Rome, focusing on the Montana family and the Church of the Sacred Light. Another unfolds in New York, centring on characters engaged in scientific and business endeavours. In Jerusalem the narrative revolves around two Orthodox Jewish families, the Kohegs and the Gudmans. Meanwhile, in Brazil, a more intricate storyline unfolds around the Santero family, the Aisen hospital, and a community associated with a neo-Pentecostal church. By the end of the story, all these narratives are united in the culmination of the end of times.

## Neo-Pentecostal everyday politics on TV

Exploring the Pentecostal revolution in Nigeria, Ruth Marshall (2009) has demonstrated how Pentecostalism can foster ‘political spiritualities’ through everyday life practices such as prayer, fasting, and church-going. Through conversion to Pentecostalism, believers engage in a moral programme of self-transformation practices – technologies of the self^3^ – including Bible study, self-examination, and public witness, which in turn provides the basis to critique the corrupt political spaces in Nigeria (Marshall 2009). In this context, individual actions are informed by Pentecostal everyday politics. As Marshall’s work demonstrates, there is a need to study politics beyond formal religious and political institutions. Instead, scholars should focus on how political ideas and religious doctrines circulate through preaching, testimonies, and, particularly, material culture. Thus, I follow her advice and analyse how the telenovela *Apocalypse* articulates this neo-Pentecostal everyday politics on TV by infusing a particular religious moral programme in its plot.

An important dimension of the emerging neo-Pentecostal culture, as articulated in Brazilian biblical telenovelas, revolves around themes related to dramatic life transformations experienced by individuals encountering the neo-Pentecostal faith. These testimonies of faith and redemption are pivotal components of Pentecostal culture, serving as a technology of self, through which believers engage in practices of self-transformation and shape their subjectivity. These narratives often portray individuals overcoming life adversities such as violence, poverty, infertility, and drug abuse, finding redemption through religious conversion and commitment.

In the telenovela *Apocalypse*, characters experience conversion to neo-Pentecostalism as part of their dramatic story arcs. For example, Osvaldo Santero, a former criminal who embraced neo-Pentecostalism while in prison, undergoes a significant transformation. He reforms his life, finds stable employment, and builds a loving family, symbolising the power of faith. Another character, Tiago Santero, Osvaldo’s son, grows up in the harsh reality of the favelas and turns to drug abuse. Osvaldo, after his conversion and release from prison, fervently prays for and evangelises Tiago, ultimately leading to Tiago’s conversion. Tiago leaves drugs behind, becomes a medical doctor, and uses his faith to influence and help his patients, including praying for them and encouraging them to embrace faith. This approach of incorporating religious testimonies into telenovela storylines offers a distinctive way to portray the power of faith and transformation within the context of popular entertainment, reaching a broader audience beyond traditional congregational settings.

Prayer is also portrayed as a mechanism through which individuals can transform their own realities and the realities of others. The telenovela has a pedagogic function, as it teaches the audience how to approach prayer in the neo-Pentecostal tradition. For instance, an ex-Catholic believer converted to neo-Pentecostalism is taught by his pastor how to pray (Episode 124). According to the pastor’s instructions, God is always able to hear us without any intermediary (i.e. a priest). Prayer should be simple, and the only thing needed is to address the prayer directly to Jesus with strong sincerity. These examples demonstrate how neo-Pentecostal themes of testimony and prayer are smuggled into the intricate plots of telenovelas. By organically incorporating these themes, neo-Pentecostal culture becomes not only more accessible but also more appealing to a broader audience.

Moreover, the Bible and Bible study are promoted throughout the plot of *Apocalypse*. The narrative underscores the significance of understanding and interpreting the Bible as a crucial skill to navigate the end times. Biblical knowledge, espoused mainly by neo-Pentecostal characters, including the pastor, becomes an inspiration for a small religious community in Rio de Janeiro during tumultuous periods of the Great Tribulation. This knowledge becomes instrumental in identifying the antichrist and discerning the subsequent stages of the unfolding end times. Verses – primarily from Revelation – punctuate the narrative, elucidating apocalyptic developments and evoking specific emotions. Events like the Rapture, when good Christians are taken up to heaven, are elucidated through the neo-Pentecostal understandings of a range of biblical verses such as Revelation 3:10; 2 Peter 3:10; Matthew 24:20–41.

The power of the Bible is accentuated on numerous occasions throughout the narrative. For example, a character suffering from depression finds comfort and healing through the Bible and Bible study (Episode 29). Moreover, the Bible serves as a guide for the main protagonist, Zoe Santero, helping her interpret reality and maintain her faith under challenging circumstances. Zoe is depicted engaging deeply with the Bible – reading, interpreting, and teaching from it at various points in the story. The portrayal of the Bible and biblical knowledge in the plot not only illustrates its practical application in one’s life, such as alleviating depression or providing guidance during tough times, but also emphasises its educational role in conveying to the audience specific biblical interpretations aligned with a particular neo-Pentecostal culture.

An important political aspect presented in the telenovela *Apocalypse* is its anti-Catholic stance. This is particularly relevant as Brazil, long recognised as one of the world’s largest Catholic nations, is undergoing a significant religious transformation. Although Catholicism remains influential, it no longer dominates the country’s spiritual landscape. Projections from the Brazilian Institute of Geography and Statistics suggest that by 2030, evangelicals may surpass Catholics in numbers, reaching 39.8% of the population compared to 38.6% for Catholics (Alves et al. 2017). While this shift is not guaranteed – especially given the Catholic Church’s active efforts to retain and grow its membership – it highlights a marked change in Brazil’s religious makeup. The anti-Catholic tropes portrayed in the telenovela both reflect and reinforce the broader religious shift in Brazil, echoing long-standing anti-Catholic sentiments found within neo-Pentecostal teachings. For example, the veneration of saints – central to popular Catholicism – is rejected and reinterpreted as demonic within the demonology of the UCKG (Oro 2005). This anti-Catholic stance is clearly mirrored in the telenovela’s narrative. The ‘Church of the Sacred Light’, represented as a proxy of the Catholic Church, is portrayed with strong anti-Catholic sentiments. A segment of the plot unfolding in Rome focuses on the notion of the antichrist being nurtured within the ‘Church of the Sacred Light’. The ‘Holy Father’, akin to the Pope, is depicted as a corrupt individual, solely power-hungry, able even to kill his adversaries. From the outset, the priest who becomes the Holy Father is depicted receiving directives straight from Satan, guiding the antichrist in executing a diabolical plan. The narrative also emphasises the dangerous nature of the cult of the Virgin of the Sacred Light (paralleling the cult of the Virgin Mary) and the church’s involvement in paedophilia. While these anti-Catholic narratives can be very controversial in a country with the largest Catholic population in the world, they are very common within neo-Pentecostal culture. These narratives are connected with the neo-Pentecostal belief in ‘spiritual warfare’, which suggests that reality is immersed in a spiritual battle where God and Satan are in constant struggle. In this framework, elements from non-Pentecostal religious traditions are demonised and persecuted, such as Catholic cults, Afro-Brazilian divinities, to name but a few. Therefore, *Apocalypse* not only educates viewers about ‘spiritual warfare’ but also instils anti-Catholic sentiments.

The telenovela employs dramatised narratives of transformation, redemption, and the importance of the Bible to propagate a neo-Pentecostal moral programme. By incorporating elements such as testimony, prayer, and Bible study into popular culture, it disseminates both neo-Pentecostal self-practices and anti-Catholic sentiments. These narratives depict conversion and prayer as life-changing forces and promote the Bible as a key to a better life and eschatological insight. Given the influential role of telenovelas in shaping Brazil’s national culture and collective imagination, as noted by Lopes (2009) and Martín-Barbero (2004), these melodramatic narratives serve as a ‘national pedagogy’ (Abu-Lughod 2004). The telenovelas thus facilitate a shared vision of a virtuous religious nation, supporting a neo-Pentecostal agenda that intertwines morality with political spiritualities and the ideal of a Christian nation purified of demonic influences. Although not as widely popular as the long-running secular telenovelas produced by TV Globo, TV Record’s efforts in creating productions like *Apocalypse* represent an attempt to craft a new national narrative that attends and reflects the religious shift taking place in Brazil, by incorporating neo-Pentecostal values into the storyline.

## Learning to love Israel and the Jewish people

Philo-Semitism is emerging as a notable element of neo-Pentecostalism in the Global South. The term can be defined as an admiration for Jewish culture and the people of the Old Testament, leading to an interest in modern Jews and Judaism and, ultimately, a desire for a symbiotic relationship with Jews and Israel (Bruder 2017). This fascination with Israel, Jews, and Judaism can manifest in various ways, including pilgrimages to the Holy Land with the intention ‘to walk where Jesus walked’ (Kaell 2014), adopting certain Jewish rituals such as celebrating Passover and Hanukkah (F. L. Shapiro 2015), and engaging in a more profound Judaising process, which involves adopting Jewish laws and fostering a Jewish identity (Carpenedo 2021).

The telenovela *Apocalypse* does not promote a comprehensive Judaising programme to its audience. However, it does incorporate philo-Semitic elements within its storyline, such as love and admiration for Israel, the Jewish people, and their material culture. A significant portion of the narrative occurs in Jerusalem and features two Orthodox Jewish families – the Kohegs and the Gudmans. In the narrative and echoing dispensational teachings, Jewish people correspond to a high tier in the religious hierarchy, as they are seen as the chosen people of God. Although the plot occasionally critiques the rigidity of Orthodox Jewish customs, these families are depicted in a favourable light as respectable and virtuous individuals. For example, in Episode 67 the value of modesty as practised by Orthodox Jewish women is celebrated. Hanna Koheg explains the importance of modesty, comparing women’s need for discretion to the Torah scroll’s requirement for careful handling and protection. In stark contrast, the telenovela casts atheism, alternative spiritualities, and Catholicism in a negative light, portraying them as targets within the ‘spiritual warfare’ framework. Atheistic beliefs are rigorously questioned, non-mainstream spiritual practices are ridiculed, and Catholicism is portrayed as perilous and satanic, especially due to its associations with the antichrist and his malevolent schemes. Despite not being portrayed as the epitome of the ‘true neo-Pentecostal faith’, Judaism and Jewish characters are shown in a friendly manner and are considered crucial allies in the fight against satanic forces. Notably, by the telenovela’s conclusion, members of the Koheg and Gudman families play a pivotal role in opposing the antichrist and in the evangelisation of Jewish people. The plot also demonstrates a fruitful partnership between Jews and neo-Pentecostals, with neo-Pentecostal characters often developing romantic relationships with Jewish individuals. A prime example is the protagonist couple: Zoe Santero, a dedicated neo-Pentecostal believer, and Benjamin Gudman, a secular Jew who later converts to neo-Pentecostalism.

A significant manifestation of philo-Semitism in the telenovela is ‘love for Israel’, a sentiment frequently evident throughout the narrative. Israel is portrayed as a land blessed by God and immersed in sacredness (Episodes 69, 70, and 71). The historical and cultural importance of Jerusalem is also highlighted: it ‘is fascinating, its history and culture’ (Episode 71:16 min). The city is also depicted as a vital spiritual place that offers deeper insights into Jesus, his teachings, and his role in salvation. For example, Benjamin, initially a secular Jew, embarks on a spiritual journey from New York to Israel, where he discovers evidence of Jesus’s existence and his salvific power. Benjamin is not alone in his quest. Many Christians travel to the Holy Land to experience its sacred sites and relics (Kaell 2014). Reflecting this allure of Israel, Brazilian Pentecostals have been organising pilgrimages to the Holy Land since the nineties, even establishing their own travel agencies to manage the growing influx of pastors and worshippers travelling from Brazil to Israel (Machado, Mariz, and Carranza 2022). M. Shapiro (2021) observes that these pilgrimages are driven by a collective belief in a divine presence that permanently resides in the Holy Land, home to God’s chosen people, the Jews. Therefore, by highlighting Israel’s sacred nature in its storyline, the *Apocalypse* telenovela not only underscores the religious significance of the Holy Land but also educates its audience to recognise and cherish this inherently sacred place.

The telenovela also displays a deep appreciation for Jewish religious practices. Throughout the series, it acts as an educational showcase where viewers are invited to admire and learn more about Jewish rituals. Key Jewish rites of passage are portrayed and explained: a baby’s circumcision (Episode 7), a teenager’s Bar Mitzvah (Episode 11), and a Jewish wedding (Episode 89) are all featured. Additionally, the celebration of Pesach by the Orthodox Jewish families is depicted and explained (Episode 91). The telenovela also illustrates everyday Jewish religious practices. It shows women reading from their *Tehillim* books with eyes closed and men chanting prayers in Hebrew with their *tallitot*, the traditional prayer shawls (Episode 69). Therefore, the notable inclusion and pedagogic explanation of Jewish rites and customs within the context of a neo-Pentecostal biblical telenovela highlights the nuanced ways philo-Semitic views are being mobilised into neo-Pentecostal culture. Thus, such melodramas contribute to fostering philo-Semitic discourses, popularising philo-Semitic sentiments to wider audiences.

Another key dimension in the production of philo-Semitic discourses in Brazilian neo-Pentecostal culture is the utilisation of Jewish material culture. The telenovela *Apocalypse* showcases this through various elements ranging from scenographic details to narrative threads. Sacred Jewish items, such as *menoroth* (multi-branched candelabras), are prominently displayed in Jewish characters’ homes. The attire of Orthodox Jewish families is authentically depicted with dark, modest clothing, with women in wigs and hats and men in *tallitot*, *payot* (side curls), *kippot* (skullcaps), and black fedoras. Less conservative Orthodox Jewish characters are shown wearing subtle star of David necklaces. Furthermore, the plot celebrates significant Jewish sacred sites, including the Kotel (Western Wall).

This prominent display of Jewish material culture in the biblical neo-Pentecostal telenovela underscores not only a reverence for Judaism and its historical significance to Christianity but also mirrors the rising philo-Semitic sentiment within Brazilian neo-Pentecostalism itself, especially evident in the UCKG and its media arm, Record TV. For over a decade, the UCKG has integrated Jewish symbols like the Star of David, the menorah, and the *tallit* into their places of worship. The pinnacle of this trend was reached in 2014 with the inauguration of a Temple of Solomon replica on the outskirts of São Paulo. This grand structure, which cost 230 million dollars, is modelled after the temple described in the Second Book of Chronicles, Chapter 2. At the extravagant inauguration ceremony, numerous Jewish symbols and items were prominently featured. UCKG founder Edir Macedo described the temple as a way to spiritually connect believers with Jerusalem (Macedo 2014). Scholars have interpreted the UCKG’s adoption of Jewish symbols – most notably the grand construction of the Temple of Solomon – as a strategic effort to consolidate its position within Brazil’s dynamic religious landscape. By establishing a material connection to Jerusalem, the church seeks to enhance its religious authenticity, symbolically transforming the outskirts of São Paulo into an extension of the Holy Land and legitimising its growing global presence. According to Topel (2011), this symbolic alignment with Judaism also serves an institutional purpose: it represents a rebranding strategy aimed at distinguishing the UCKG’s ‘religious product’ in an increasingly competitive market. In a similar vein, Lehmann (2020) argues that the use of Jewish-themed imagery is less about theological commitment and more about constructing a unique institutional lineage, reinforcing the church’s identity without necessarily adhering to a cohesive doctrinal framework. Regardless of intent, philo-Semitic sentiments are being actively mobilised in various forms within neo-Pentecostal material culture in Brazil. Therefore, the mobilisation of philo-Semitic sentiments within the *Apocalypse* telenovela can be seen as part of a broader moral and political project within UCKG neo-Pentecostal culture. By highlighting the sacredness of Israel and educating audiences about Jewish rituals, customs, and material culture, the plot of *Apocalypse* not only instructs viewers about Judaism but also instils love and admiration for Israel and the Jewish people. Thus, the popularisation of philo-Semitic narratives through these biblical telenovelas demonstrates how specific political spiritualities can be fostered through popular neo-Pentecostal culture.

## Mobilising Christian Zionist sentiments through melodrama

Although Christian Zionism is a multifaceted phenomenon with distinct characteristics (Freston 2020), it can be understood as the importance given by Christians to Jews returning and living in Israel. This importance is based on the belief that the covenant God made with Jews is eternal and that Israel works as God’s prophetic clock able to predict the second coming of Christ. This emphasis on Jews returning to Israel is translated into strong geopolitical activism in favour of the State of Israel.

A good example of the influence of Christian Zionism is US contemporary foreign policy. Anchored on Genesis 12:3, Christian Zionists believe that the United States is blessed because it has been protecting Israel and the Jewish people (Spector 2009). In 1980 Jerry Falwell claimed that America was a blessed nation because it had blessed the Jewish people, and if the US wants to keep its protagonism in the world, it needs to continue to protect Israel (Spector 2009). These ideas had an important impact on George Bush’s administration (2001–2009), which promoted unconditional support for Israel, stressing the role of the ‘promised land’ and the Jewish return to Israel as a prophetic sign, thus favouring Israel over Palestine in the geopolitical arena (Spector 2009). Likewise, Trump was not only the first US president to visit the Western Wall but also moved the embassy from Tel Aviv to Jerusalem. Therefore, Christian Zionists have influenced and developed their own ‘evangelical foreign policy’ in the US (Sturm 2017). At the grassroots level of churches, this activism can be translated through prayer chains for the State of Israel and its defence forces and financial aid to educational and medical initiatives in the West Bank (Phillips 2009). For instance, in 2009 the organisation Christians United for Israel raised $58 million for Israel and its settlements (Sturm 2017).

The plot of *Apocalypse* shows Christian Zionist themes and political inclinations in diverse ways. For instance, geopolitical support for Israel and awe of its military capabilities are portrayed in different forms in the telenovela. An important theme highlighted is the admiration transmitted towards Israel and its military power. Throughout the plot taking place in Jerusalem, viewers are introduced to young and good-looking Israeli soldiers (women and men) proudly showcasing their large weapons, ‘Tavor’ TAR-21, in the background of the scenes. The plot frequently highlights the formidable defence capabilities of the Israeli army with statements like ‘Israel has the best army in the world’ and ‘The Israeli army is one of the most feared and respected in the world’ (Episode 66:39). Amidst a war initiated by the antichrist, a character asserts ‘Israel knows how to protect itself’, and praises the effectiveness of Israel’s missile defence system (Episode 100:37). This admiration for Israeli militarism extends beyond the telenovela, mirroring a common narrative among neo-Pentecostal churches that celebrate Israel’s technological advancements and military triumphs (Machado, Mariz, and Carranza 2022). By mobilising discourses emphasising Israel’s military power, telenovelas transmit not only the idea that Israel is a powerful nation but also that its military success can be read as God’s blessing.

The telenovela also features a compelling character, Noah Koheg, who embodies tropes beloved in melodrama and Christian Zionism. As a central figure, Noah serves in the Israeli army despite being from an Orthodox Jewish family – a notable departure from reality since Orthodox Jews typically are exempt from military service in Israel due to an agreement that allows them to dedicate their lives to Torah study. This plot discrepancy enhances the narrative’s appeal by merging neo-Pentecostals’ reverence for Orthodox Jews (Carpenedo 2021) with the valorisation of Israeli soldiers. Noah is portrayed as a quintessential hero through various Hollywood-style scenes: he discovers explosives in a city, frustrating an orchestrated terrorist attack (Episode 17); stops a terrorist on a Jerusalem school bus, thereby saving children onboard (Episode 20). When he is recognised as a saviour by a child from the bus, the girl is initially frightened by his gun. However, Noah assures her that it is for defence, reinforcing the theme of Israel’s right to self-protection. In another instance, Noah eliminates terrorists during an attack in Jerusalem, and is hailed as a hero by the crowd (Episode 31). Thus, by merging Orthodox Jewish faith with the excellence of the Israeli military, Noah symbolises both religious devotion and militaristic strength, capturing Christians’ fascination with Jews and Judaism, and support for the Zionist cause, as he defends the Holy Land from terrorists. This emphasis on militaristic tropes and fascination with weapons mirrors broader trends found in the Brazilian evangelical right. The evangelical Christian right often promotes pro-military agendas and supports tough public security policies as responses to urban violence. In this context, they oppose stricter gun control measures and instead advocate for gun rights, framing access to firearms as essential for both personal and community protection (Almeida 2017).

Beyond the antichrist and his actions behind the conflicts portrayed in the plot, the enemies are referred to as Arabs, Palestinians, Muslims, the Islamic State, and terrorist groups such as ISIS and Hezbollah. For instance, in episode 82 we see terrorist attacks carried out by Palestinians under the instruction of the antichrist, killing many Jews while they pray at the Kotel. The consequences of these attacks are dramatic, with one of the fatalities being Noah’s biological mother. Later that day, disturbed by such violence, Noah’s sister, Hannah, exclaims: ‘ISIS, Hezbollah, and the Islamic State are insane for daring to attack Israel’s most sacred site, the Kotel!’ The depiction of Muslims as adversaries continues in the plot of *Apocalypse*. In episode 100, following the summoning of the red horseman who announces war (Revelation 6:3–4) with the antichrist’s support, Muslims are blamed for initiating the bloody Third World War. The telenovela *Apocalypse* thus depicts Arabs and Muslims as aligned with satanic forces and as enemies of both Jews and neo-Pentecostal Christians. Consequently, these perspectives offer the audience a spiritual dimension of current conflicts in the Middle East. This representation echoes the beliefs of North American Christian Zionists, who interpret the conflicts in the Middle East not merely as a struggle against terrorism or a clash of civilisations but as a spiritual battle where Muslims are seen as the army of Satan (Spector 2009).

While Arabs and Muslims are demonised in the plot of *Apocalypse*, the need for Jews to stay in Israel and protect the land is reinforced as Israel has a pivotal role in God’s plan. For instance, when the Third World War began, part of Koheg’s family was abroad, and they promptly wished to return to Israel. Being asked why they were eager to return to the conflict’s epicentre, one family member explained that ‘Jews must stay in Israel’, since Jews have endured numerous persecutions, such as during the Holocaust, and that ‘God had given them this land’ and they ‘must fight for Israel’ (Episode 101:34). In doing so, the narrative further reinforces the core themes of Christian Zionism, including the belief that the Jewish settlement in Israel is biblically sanctioned. This view, influenced by dispensationalism, posits that the Jewish return to the land of Israel is a prerequisite for the second coming of Christ and the establishment of the Kingdom. Thus, the telenovela portrays the necessity for Jews to return, remain, protect, and be prepared to defend Israel in the unfolding end of times.

A central theme in dispensationalist thought – the eventual conversion of Jews to Christianity – is also reflected in the plot of the telenovela. The dispensationalist prophecy embraced by the TV show conveys that Jews will return to the Holy Land and establish a state without accepting Jesus. However, following the Rapture of the church and the onset of the Great Tribulation – including the reign of the antichrist – Jews will gradually start accepting Jesus as their saviour and become the principal evangelists of the millennial era. In the *Apocalypse* telenovela, this dispensationalist teaching unfolds through the Orthodox Jewish families of the Kohegs and Gudmans. Initially they resist conversion to Christianity, as Jesus did not fulfil the Messianic prophecies of Judaism – for example, Jesus is not a descendant of David and cannot be the son of God, since God does not take physical form in Judaism (Episode 94). However, as the plot progresses, Noah Koheg and Saulo Gudman are persuaded to accept Jesus as their saviour by the two witnesses, Enoch and Elijah (as mentioned in Revelation 11:3–13), after witnessing a series of miracles they perform, such as turning the sea into blood (Episode 72). After converting to Christianity, Noah and Saulo abandon their Orthodox Jewish attire and declare that faith is far more important than religion and tradition (Episode 116). Inspired by the conversion stories of Noah and Saulo, both the Koheg and Gudman families fully embrace Christianity. In contrast, those Jews who do not accept Jesus are deceived by the antichrist and begin to worship him as the false Messiah.

Later in the plot, and reflecting dispensational teachings, Noah and Saulo come to understand that they are among the 144,000 Jews chosen by God to bear witness and evangelise to other Jews (Episode 128). According to dispensationalist eschatology, during the Great Tribulation 144,000 Jews − 12000 from each tribe – will recognise the truth of the Christian message, accept Jesus as their saviour, and begin to spread the gospel among Jews (Ariel 2000). These Jews, now followers of Yeshua (Jesus), refuse to accept the mark of the beast and are consequently persecuted by the antichrist (Episode 125). They form an armed resistance, combining aspects of Israeli militarism with the ethos of neo-Pentecostal Bible study groups. Under God’s protection, this small Israeli army miraculously escapes death and destruction and withstands the forces of evil.

The exploration of tropes surrounding the conversion of Jews to Christianity – and the corresponding condemnation of those who refuse – reveals how the dramatisation of dispensational teachings can mobilise particular attitudes towards Jews that can go beyond philo-Semitism. The concept of allosemitism, as developed by Bauman (1998), offers a useful lens for understanding the complex and ambivalent ways Jews have been perceived. Rather than viewing philo-Semitism and anti-Semitism as opposing forces, Bauman argues that they are both expressions of the same underlying logic: the construction of the Jew as radically ‘other’. Allosemitism encompasses both positive and negative attitudes, portraying Jews as fundamentally distinct from the rest of society – admired, feared, or resented, but always set apart. This essentialising perspective disconnects the symbolic figure of ‘the Jew’ from real Jewish individuals and communities, allowing for projections that can shift easily from fascination to hostility. Bauman warns that even seemingly benign or admiring views can reinforce exclusion and lay the groundwork for anti-Semitic violence. By maintaining the Jew as an exceptional figure – whether venerated or vilified – allosemitism sustains a logic of otherness that can ultimately lead to marginalisation, scapegoating, and, in its most extreme form, the desire for elimination.

Therefore, the prominent inclusion of tropes such as the return and settlement of Jews to the Holy Land, the exceptionalism of the ‘God-blessed’ Israeli army, the demonisation of Arabs and Muslims, and the evangelisation of Jews exemplify the ways dispensational and Christian Zionist teachings are woven into biblical telenovelas like *Apocalypse*. This illustrates the utilisation of Christian Zionism as a central political-spiritual value within the emerging neo-Pentecostal political culture.

## Conclusion

This contribution indicates how political ideas circulate through religious material culture such as Brazilian biblical telenovelas. By exploring the emergence of a particular religious moral programme endorsing neo-Pentecostal testimonies, prayer, and Bible study, the contribution demonstrates how political spiritualities could be fostered through popular neo-Pentecostal culture. In depicting the Christian ‘love for Israel’ and the ‘Jewish people’ as well as clear support for the State of Israel, its settlements, and militarism, my analysis of the telenovela *Apocalypse* also shows how Christian Zionist and philo-Semitic sentiments can be popularised and transmitted to mass audiences. Furthermore, the UCKG choice to air this telenovela again amid the COVID-19 pandemic and the rising tensions in the Middle East is significant. It points to a purposeful strategy of framing these events using apocalyptic imagery – tying war and the pandemics to prophecies of the end times in a manner that reinforces the series’ theological messaging.

As demonstrated, biblical telenovelas like *Apocalypse* function as melodramatic narratives and communicative resources for shaping a ‘narrative of the nation’ (Lopes 2009), activating viewers’ religious imagination while instilling specific political inclinations. These productions operate as cultural and political forces, mobilising theological themes to engage with both national discourse and global religious imaginaries. For instance, by portraying Muslims as aligned with satanic forces, telenovelas like *Apocalypse* offer audiences a framework for interpreting contemporary conflicts in the Middle East, reinforcing binary moral worldviews. Central to this dramatic narrative is a pro-Israel stance, often framed through spiritual and prophetic lenses, which reflects a broader political tendency aligned with conservative Brazilian nationalism. This alignment became especially pronounced during Jair Bolsonaro’s presidency, characterised by strong diplomatic ties with Israel and enthusiastic support from evangelical religious leaders. Together, these elements illuminate the emergence of a distinct neo-Pentecostal political culture in the Global South – one defined not only by an everyday moral agenda but also by pronounced Zionist and philo-Semitic commitments.

## Notes

1. No ethical approval was required for this study as it was based solely on desk-based research using publicly available sources. According to the guidelines of the University of Groningen, such research does not fall under the remit of institutional ethical review.

2. ‘I will bless those who bless you, and whoever curses you I will curse; and all peoples on earth will be blessed through you.’

3. ‘Technologies of the self’ refer to specific operations performed by individuals on their ‘own bodies, souls, thoughts, conduct, and way of being’ (Foucault 1997, 242). These operations seek the transformation of oneself to attain a state of happiness or purity. For Pentecostals, this transformation signifies becoming moral subjects aligned with Pentecostal values.
